# A Novel Discovery: Holistic Efficacy at the Special Organ Level of Pungent Flavored Compounds from Pungent Traditional Chinese Medicine

**DOI:** 10.3390/ijms20030752

**Published:** 2019-02-11

**Authors:** Zhao Chen, Yanfeng Cao, Yanling Zhang, Yanjiang Qiao

**Affiliations:** 1 School of Chinese Materia Medica, Beijing University of Chinese Medicine, Beijing 102488, China; zhaochen@bucm.edu.cn (Z.C.); shu_nirvana@sina.com (Y.C.); 2 State Administration of Traditional Chinese Medicine, Research Center of TCM-Information Engineering, Beijng 102488, China

**Keywords:** TCM, pungent flavored compound, cardiovascular disease, liver disease, systems pharmacology, blood-activating and stasis-resolving, organ location

## Abstract

Pungent traditional Chinese medicines (TCMs) play a vital role in the clinical treatment of hepatobiliary disease, gastrointestinal diseases, cardiovascular diseases, diabetes, skin diseases and so on. Pungent TCMs have a vastness of pungent flavored (with pungent taste or smell) compounds. To elucidate the molecular mechanism of pungent flavored compounds in treating cardiovascular diseases (CVDs) and liver diseases, five pungent TCMs with the action of blood-activating and stasis-resolving (BASR) were selected. Here, an integrated systems pharmacology approach is presented for illustrating the molecular correlations between pungent flavored compounds and their holistic efficacy at the special organ level. First, we identified target proteins that are associated with pungent flavored compounds and found that these targets were functionally related to CVDs and liver diseases. Then, based on the phenotype that directly links human genes to the body parts they affect, we clustered target modules associated with pungent flavored compounds into liver and heart organs. We applied systems-based analysis to introduce a pungent flavored compound-target-pathway-organ network that clarifies mechanisms of pungent substances treating cardiovascular diseases and liver diseases by acting on the heart/liver organ. The systems pharmacology also suggests a novel systematic strategy for rational drug development from pungent TCMs in treating cardiovascular disease and associated liver diseases.

## 1. Introduction

Cardiovascular disease (CVD) is the leading cause of mortality worldwide, with ischemic heart disease, heart failure, and stroke accounting for the majority of deaths [1,2,3,4]. Cardiovascular disease progresses by biological processes in the arteries and heart that eventuate in myocardial infarction, stroke, heart failure, and other vascular morbid events [2,3].

Vegetarian dietary patterns reduce CVD mortality and the risk of coronary heart disease (CHD) by 40% [1]. Plant-based diets are the only dietary pattern to have shown a reversal of CHD [1]. Traditional Chinese medicines (TCMs) are natural source herbal medicines, and Chinese ancient medical practitioners gradually comprehended the influence that TCMs (several plants and animals) exerted on the human body during the long-term practice. TCM plays an important role in the treatment of cardiovascular diseases.

Modern pathology shows that blood stasis is generally manifested by cardio-cerebrovascular diseases, such as myocardial infarction, coronary heart disease, and high blood pressure, resulting from hematological disorders including hemorrhage, congestion, thrombosis, and local ischemia [5,6]. Blood-activating and stasis-resolving (BASR) TCMs are mainly used to treat blood stasis syndrome. They have been widely applied in treating cardiovascular disease [7,8,9,10] in modern science. BASR herbs [11,12,13] are mainly include *Chuanxiong Rhizome* (Chinese name Chuanxiong), *Carthami Flos* (Chinese name HongHua), *Radix Salviae* (Chinese name DanShen), *Radix Paeoniae Rubra* (Chinese name ChiShao), *Curcumaelongae Rhizoma* (Chinese name JiangHuang), *Dalbergiae Odoriferae Lignum* (Chinese name JiangXiang), *Foeniculi Fructus* (Chinese name XiaoHuiXiang), *Angelicae Sinensis Radix* (Chinese name DangGui), and *Panax Notoginseng* (*Burk.*) *F. H. Chen Ex C. Chow* (Chinese name SanQi), etc. These BASR TCMs mainly exert therapeutic effects by improving cardiovascular activities [14,15]. They also have been widely applied routinely in clinical practice for treating coronary heart disease [10,12,15,16], cardio protection [17,18,19], anginapectoris [20,21], hypertension [22], and hyperlipidemia [23], etc.

Chronic liver disease (CLD) is a major cause of mortality, morbidity, and health care resource utilization worldwide [24]. From 1980 through 2010, mortality related to CLD increased by 46% worldwide [25]. WHO estimates that liver cancer is responsible for around 47,000 deaths per year in Europe. Liver disease affects young and middle-aged people, and in terms of years of life lost (YLL), liver cirrhosis is the 13th leading cause globally, the sixth leading cause of YLL in the developed world, and the eighth leading cause in Western Europe [25]. The incidence and prevalence of cirrhosis and primary liver cancer are key to understand the burden of liver disease [26].

Harmful alcohol consumption, viral hepatitis B and C, and metabolic syndromes related to overweight and obesity are the leading causes of cirrhosis and primary liver cancer in Europe [27]. Available data suggest the prevalence rate of non-alcoholic fatty liver disease (NAFLD) is 2 to 44% in the general European population (including obese children) and 42.6 to 69.5% in people with type 2 diabetes [27]. The hospitalized cases and mortality from alcoholic liver disease (ALD) are increasing in Taiwan and worldwide. Meanwhile, the Asia Pacific region also has a high prevalence of hepatitis B virus (HBV) and hepatocellular carcinoma (HCC) [27]. Chronic hepatitis B affects 0.5 to 0.7% of the European population. In the last decade, the prevalence of chronic hepatitis C was 0.13 to 3.26% [27].

Non-alcoholic fatty liver disease and nonalcoholic steatohepatitis can lead to advanced liver disease [28]. NAFLD is a common cause of chronic liver disease, and its worldwide prevalence continues to increase with the growing obesity epidemic [29].

Non-alcoholic steatohepatitis (NASH) has a potentially progressive course leading to liver fibrosis, cirrhosis, hepatocellular carcinoma (HCC), and liver transplantation [30,31]. The global epidemic of obesity has been accompanied by a rising burden of NAFLD, with manifestations ranging from simple steatosis to non-alcoholic steatohepatitis, potentially developing into hepatocellular carcinoma [32]. NAFLD is a leading cause of end-stage liver disease, hepatocellular carcinoma, and liver transplantation worldwide [33,34,35,36]. The prevalence of NAFLD is increasing at approximately the same rate as obesity. NASH and most importantly, fibrosis severity have been strongly implicated in the long-term prognosis of NAFLD patients [37,38,39,40].

To make traditional Chinese medicine (TCM) serve people all over the world better and accelerate the promising TCM-based new drug development, it is necessary to bring the ancient practice of (TCM) into line with modern standards world [41,42,43,44,45,46], among which the elucidation of the molecular mechanisms of TCM is one of the most important issues. Specifically, the material basis and the molecular mechanism of pungent flavor is not yet clear.

**Figure 1:**
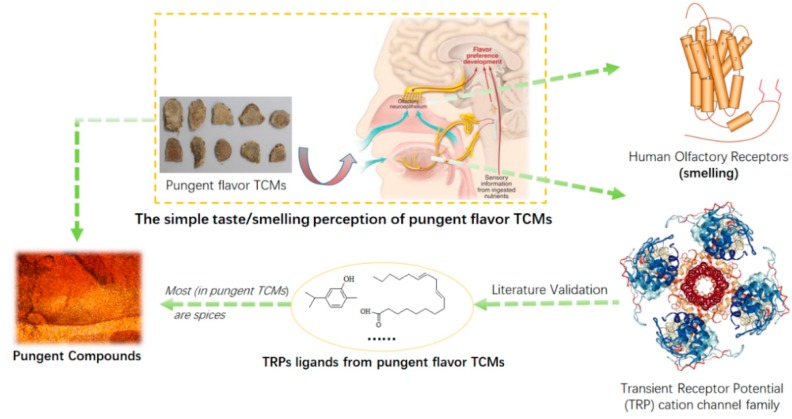
Process of discovering the pungent flavored materials from traditional Chinese medicines (TCMs).

In our recent research, we found that the pungent TCMs have the vastness of spices. Moreover, we would elucidate the material basis of pungent flavor based on the spicy compounds and the molecular mechanism of pungent flavor based on the transient receptor potential (TRP) cation channel family (Figure 1). The pungent flavor profiles of pungent TCMs always have a fragrant smell and pungent taste (Table 1). Each pungent TCM contains at least one spice or pungent flavored compound. Spices are defined as any dried, fragrant, aromatic vegetable or plant substance that contributes flavor in a whole, broken, or ground form with a strong taste [47,48]. Spices also have been used for thousands of years to enhance the flavor, color, and aroma of food [9]. In addition, they are known for their preservative and medicinal value [49,50]. In the past three decades, it has been experimentally documented that several common spices can also exert beneficial health physiological effects [51]. The antioxidant properties of herbs and spices are of particular interest given the impact of oxidative modification of low-density lipoprotein cholesterol in the development of atherosclerosis [52]. Consumption of garlic or garlic oil has been associated with a reduction in total cholesterol, low-density lipoprotein (LDL) cholesterol, and triglyceride levels [52]. Studies suggest that an intake of between half and one garlic clove per day can reduce cholesterol by 9% [53,54]. 

In worldwide studies, spices have been linked to the prevention and treatment of chronic conditions such as heart disease, cancer, Type II diabetes, and Alzheimer’s [9]. Spices are described as possessing medicinal properties, such as being anti-oxidant, antibacterial, antithrombotic, anti-atherosclerotic, hypolipidemic, hypoglycemic, anti-inflammatory, and antiarthritic, etc. [50,51,52,53,54,55]. The Spices have always been used as medicine because they are natural products easily absorbed by our bodies and generally do not have any adverse effects [49,56]. 

Herbal remedies are an important source for the discovery of new antibiotics and numerous studies have identified compounds within herbal plants that are effective [49,57,58,59]. Specifically, the pungent TCMs have numerous spices that have a great potential to be developed as new and safe agents. 

Spices in food and beverages and compounds in tobacco smoke interact with sensory irritant receptors of the transient receptor potential (TRP) cation channel family [60]. The TRP channels are polymodal transducers accepting a multiplicity of exogenous and endogenous chemicals as agonists [61,62,63]. This pungency is attributable to chemicals that activate a specific ion-channel protein, known as transient receptor potential ankyrin 1 (*TRPA1*) [64,65,66,67]. *TRPV1* (vanilloid type 1), *TRPA1* and TRPM8 (metastatic 8) not only elicit action potential signaling through trigeminal nerves, eventually evoking pungent or cooling sensations, but by their calcium conductance, they also stimulate the release of calcitonin gene-related peptide (CGRP) [60]. Moreover, *TRPA1* channels are activated by pungent compounds found in garlic, onion, mustard, and cinnamon extracts [68].

In the research field of pungent flavor in TCM terms, the ligands of TRPs ion channels have potential efficacy in cardiovascular diseases. For example, Capsaicin (highly pungent, highly volatile with a pungent odor) is a naturally occurring compound found in many pungent TCMs (*Alpiniae Officinarum Rhizoma*, *Capsici Fructus*, *Bupleuri Radix*, *Ginkgo Nut*), and it can activate the TRPV1 ion channel [69]. Eugenol (Spicy, pungent taste) is a colorless to pale yellow, aromatic oily liquid extracted from some pungent TCMs (*Cinnamomi Ramulus*, *Caryophylli Flos*, *Zingiber Rhizoma Recens*), and it can activate *TRPV1* [70,71], *TRPM8* [71], *TRPA1* [71], and *TRPV3* [72,73,74]. Carvacrol (characteristic pungent, warm odor) is a natural aromatic hydrocarbon that occurs in many pungent TCMs (*Angelicae Sinensis Radix*, *Carthami Flos*, *Moslae Herba*), and it can activate *TRPA1* [75,76], TRPM7 [77], *TRPV3* [73].

To clarify molecular mechanisms of pungent flavor for complex related diseases from a system level, the following numerous issues need to be solved urgently: (1) Which compounds are the material base of the pungent flavor in the pungent TCMs? (2) And how the pungent flavored compounds from pungent TCMs act on the heart heart/liver to treat CVDs and liver diseases clinically? (3) Which pungent flavored compounds are involved in the regulatory processes of the formulae in CVDs and liver diseases? (4) Which targets are modulated by the pungent flavored compounds to achieve the therapeutic effect? (5) Which pathologic processes are regulated by the pungent flavored compounds and herbal medicine to treat CVDs and liver diseases simultaneously? (6) Whether the pungent flavored compounds could treat CVDs and liver diseases simultaneously? In this research, the pungent flavored compounds’ holistic efficacy was mapped into the liver/heart organ by the pungent flavored compound targets. How the pungent flavored compounds from BASR TCMs treat CVDs and liver diseases by acting on the liver /heart organ is explained. The function of pungent flavor in pungent TCM is not so easily understood, and many further studies need to be considered for drug development based on the function of pungent flavor in TCM.

## 2. Results

### 2.1. Collecting the Compounds of Pungent TCMs

We collected 1159 compounds of pungent TCMs (see Table 2 for further details) and identified 39 pungent flavor compounds (see Table 3 for further details of pungent compounds). 

### 2.2. Pungent Flavor Compound-Target Network

In the pungent flavor compound-target network, *NFE2L2* (Nuclear factor erythroid 2-related factor 2) was the target with the highest degree (DD = 16), followed by AR (Androgen Receptor, DD = 15), *PTGS2* (Prostaglandin G/H synthase 2, DD = 13), *TNF* (Tumor necrosis factor, DD = 13), *NFKB1* (Nuclear factor NF-kappa-B p105 subunit, DD = 12), PPARD (Peroxisome proliferator-activated receptor delta, DD = 12), *CYP1A1* (Cytochrome P450 1A1, DD = 12), *JUN* (Proto-oncogene c-JUN, DD = 11) and etc. 

Evidence suggests that the pungent flavored compounds in pungent TCMs can act on these targets, thus, contributing to the therapeutic effect on cardiovascular diseases and liver diseases. For example (as shown in Table 4), (1). *NFE2L2* mediates activity in several organs, including those in the kidney. The transcription factor Nrf2, encoded by *NFE2L2* gene is a key regulator of cellular defense against oxidative and electrophilic stress, also governing the expression of many phase II detoxification enzymes [78]. Nrf2 is mainly expressed in hepatocytes [79] and could protect against ethanol-induced liver injury [80]. *NFE2L2* is an important component in antioxidant defenses in cardiovascular diseases such as atherosclerosis [81,82,83], hypertension [84,85,86,87], and heart failure [88,89,90,91]. Moreover, *NFE2L2* is important to vascular integrity and long-term endothelial function, for example, sustained release of NO and protection from apoptosis [92,93,94,95,96]. Laminar blood flow promotes antiatherogenic activation of *NFE2L2*, and oscillatory blood flow suppresses *NFE2L2* activation, creating a proatherogenic environment [94,97]. Nrf2 is also an important factor in the modulation of response to oxidative stress and inflammation, and both processes playing a role in liver fibrogenesis [78,98,99]. Expression levels of Nrf2 may be decreased in end-stage liver disease [78]. Additionally, there have not been clinical trials on the effects of Nrf2 activation on liver injury and disease [100,101].

(2) Androgen receptor (*NR3C4*, or AR) is another important steroid hormone receptor that is activated by endogenous androgens, mainly testosterone and 5α- dihydrotestosterone (5α-DHT). The androgen receptor (AR), also known as *NR3C4* (nuclear receptor subfamily 3, group C, member 4), is a ligand-dependent transcription factor (TF) belonging to the steroid hormone receptor (SR) group of the nuclear receptor (NR) superfamily. In humans, this superfamily contains 48 NRs. The human AR gene is located on the X chromosome [102,103,104]. As a sex hormone receptor, AR mediates the physiological actions of androgen, which is responsible for male sexual differentiation and pubertal changes. Accordingly, AR is mainly expressed in androgen target tissues, such as the reproductive tissues (i.e., prostate, seminal vesicle, epididymis, and testes), skeletal muscle, liver [105,106], and central nervous system (CNS) [107]. Androgens play a role in inducing vascular calcification through the AR [108]. ARs have illustrated clinical consequences that further explain the conflicting data on male hypogonadism and cardiovascular disease [109,110]. 

(3) Cytochrome P450 1A1 (*CYP1A1*), The cytochrome P450 (CYP) monooxygenase system is a superfamily of enzymes, which play a central role in the etoxication and metabolic activation of xenobiotics [111,112]. The highest expressions of CYP 450 are generally found in liver tissue [113], but the distribution of particular CYPs varies [114], which indicates that the actual efficiency of a drug is likely to depend on CYP expression in the target tissue. *CYP1A1* is one of the most important enzymes implicated in the metabolic activation of carcinogens. Cytochrome P450 enzymes could also express in the heart and could have an important role in the metabolism of endogenous compounds [115]. *CYP1A1* could protect against NAFLD pathogenesis [80,81,116,117]. *CYP1A1* and *CYP1A2* also play a protective role in liver damage caused by 2, 3, 7, 8-tetracholorodibenzo-p-dioxin (TCDD) and bile duct ligation (BDL) [118]. Many findings indicated that the small compounds could enhance hepatic cytochrome P450 1A1 mRNA expression in liver in animals [119,120,121,122,123,124,125]. Many researches have also shown that enzymatically functional *CYP1A1* appears to be expressed in human livers [126,127,128].

**Figure 2:**
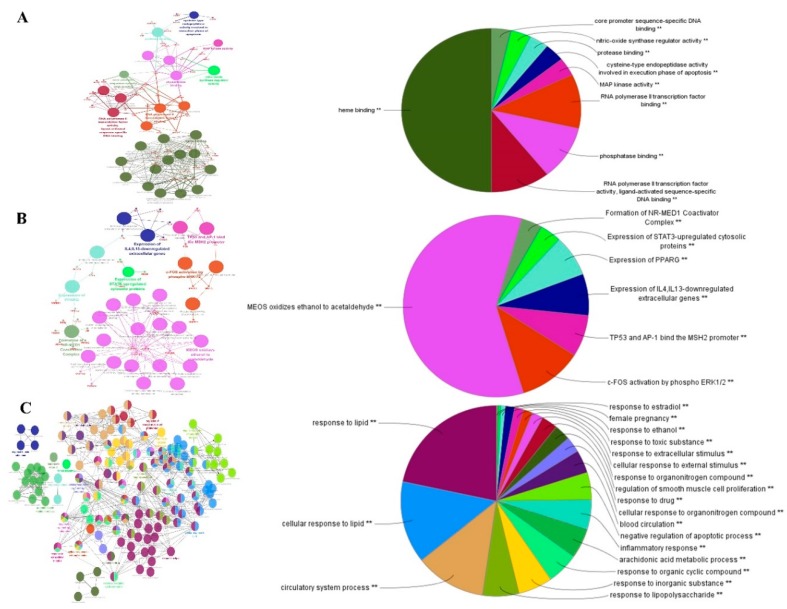
ClueGO analysis of the pungent flavored compound targets. A functionally grouped network of enriched categories was generated for the target genes. Gene ontology (GO) terms are represented as nodes, and the node size represents the term enrichment significance. Functionally related groups partially overlap. The node pie charts represent the molecular function, immune system processes, reactome analysis of targets. Only the most significant term in the group was labeled. (**A**) Representative molecular function interactions among targets. (**B**) Representative reactome analysis interactions among pungent flavored compounds’ targets. (**C**) Representative biological process among pungent flavored compounds’ targets.

To decipher the action mechanism of 81 targets associated with the cardiovascular system, ClueGO, a widely used Cytoscape plugin, was applied to identify biological interpretation and interrelations of functional groups in biological networks [126,127,128]. ClueGO creates, first, a binary gene-term matrix with the selected terms and their associated genes. Based on this matrix, a term–term similarity matrix is calculated using chance-corrected kappa statistics to determine the association strength between the terms [129]. The size of the nodes reflects the enrichment significance of the terms. As shown in Figure 2, the results were divided into three stratums: molecular functions; the reactome analysis, and biological process. Specially, the molecular functions mainly consisted of five groups: (1) heme binding; (2) RNA polymerase II transcription factor activity, ligand-activated sequence-specific DNA binding; (3) phosphatase binding; (4) RNA polymerase II transcription factor binding, and (5) MAP kinase activity, which indicated that most potential targets were related to heme binding and MAP kinase activity (Figure 2A). The reactome of the targets were mainly related to MEOS oxidizing ethanol to acetaldehyde, *c-FOS* activation phosphor *ERK1/2*, *TP53* and *AP-1* bind the *MSH2* promoter, Expression of *IL4*, *IL3*-downregulated extracellular genes, express of *PPARG* (Figure 2B). These biological functions have been linked to response to lipid, cellular response to lipid, blood circulation, circulatory system process, negative regulation of the apoptotic process (Figure 2C). Finally, we found that most of the targets were related to the activation of MAP kinase, heme binding and regulation of inflammatory factors.

### 2.3. Organ Targets Location Map

We applied systems-based analysis to introduce a compound-target-pathway-organ network that elucidated the pungent flavored compounds’ holistic efficacy at special organ level and mechanisms of pungent flavored compounds effect in treating cardiovascular diseases and liver diseases. To further explore the underlying mechanisms of pungent flavored compounds that provide therapeutic effects in CVDs and liver diseases, we studied the organ target location based on tissue expression profile and phenotypic effects. 

**Figure 3:**
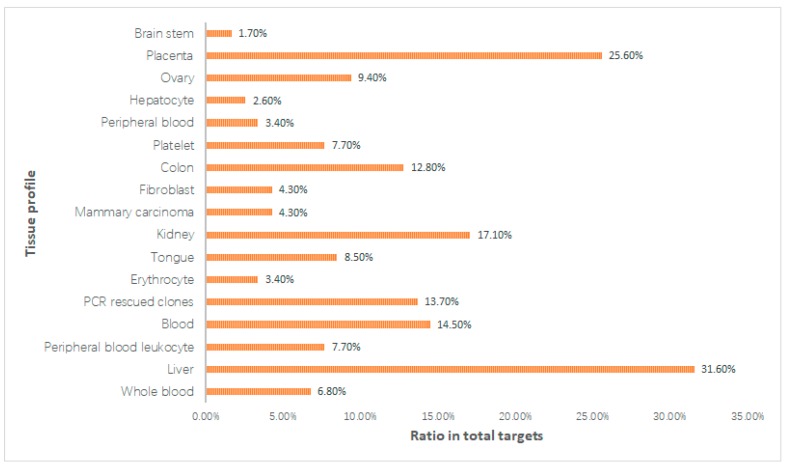
The target tissue distribution based on tissue expression profile.

We enriched the overrepresented gene ontology (GO) terms and checked the tissue distribution of the obtained targets. For GO analysis, the biological process of GO vocabulary (GOBP) was identified through GOBP terms by the DAVID database (https://david.ncifcrf.gov/) [130], and GOBP terms with adjusted *p*-values < 0.001 were observed. The target tissue distribution was determined based on the microarray analyses data of different tissue types lodged in the BioGPS bank (accessible at http://biogps.org) [131,132]. Subsequently, we mapped these 117 targets related to the cardiovascular system into the DAVID database (https://david.ncifcrf.gov/) [130] to identify the significant tissues location. The tissues location map (Figure 3) showed that 31.6% of the targets contain higher mRNA expression in liver tissue than other tissues.

**Figure 4:**
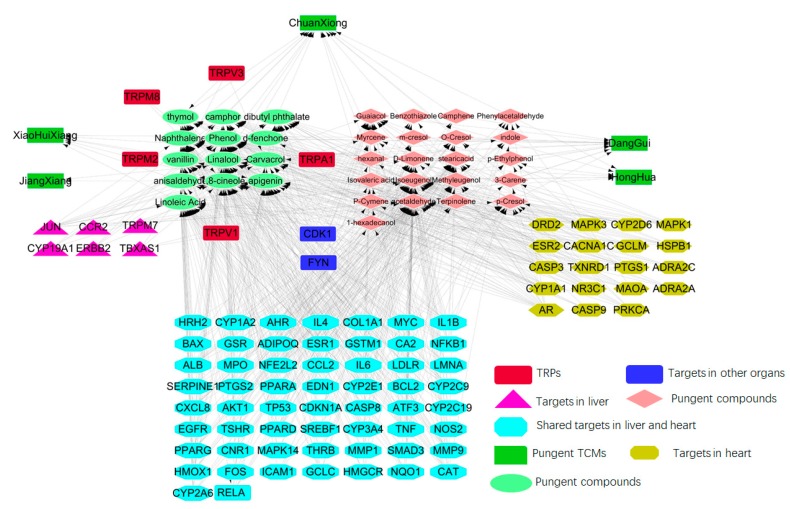
Target organ location map. The green diamond represents 5 pungent TCMs, and purple–red node represents the pungent flavored compounds. The pink triangle, yellow-green hexagon represents the target nodes along with the organs (Liver, heart) in which the target is located. The cyan ellipse represents the shared targets in liver and heart.

To understand how the multi-organs respond to indications may facilitate the development of enhanced detection and treatment modalities for complex disease on a system level, the target organ location map was constructed (Figure 4). As shown in Figure 4, 59 shared targets of the liver and heart organ were identified. Therefore, shared targets of the heart and liver could provide novel target potential for the treatment of CVDs and liver diseases. If two diseases share a large number of disease genes, the disease pairs become more comorbid, and they are closely associated [133].

Eighty-one targets related to cardiovascular system were mapped into 125 organs at different levels according to the Gene ORGANizer database (accessible at http://geneorganizer.huji.ac.il/) [134]. Gene ORGANizer is a phenotype-based tool that directly links human genes to the body parts they affect, and it provides partial or skewed information on the whole organs. Gene ORGANizer can analyze gene lists to test whether they are enriched. Thus, Gene ORGANizer is a tool that enables researchers to analyze associations with organs in a genome-wide context. Third, Gene ORGANizer focuses on whole organs (e.g., heart), systems (e.g., the cardiovascular), or anatomical regions (e.g., the thorax), rather than on specific cell types or tissues (e.g., cardiomyocytes) based on expression analyses [134]. This research explored the potential mechanism of pungent flavored compounds in the treatment of cardiovascular diseases and liver diseases. More importantly, we compared the organ patterns based on the distribution of pungent flavored compound targets in the heart and liver. Based on the target phenotype pattern, the organ targets location map was divided into two organ modules, namely liver organ and heart organ. The organ distribution of 81 pungent flavored compound targets in cardiovascular system is shown in Figure 4.

Therefore, these 62 liver high-abundant targets are considered as the liver organ, accounting for 76.5% of 81 targets related cardiovascular system. There are 77 targets (accounting for 95.1% of 81 targets related cardiovascular system) located in the heart, they are potentially effective targets for the treatment of CVDs. In addition, there are 59 targets shared between liver organ and heart organ. Most targets acted on both liver organ and heart organ, which suggests that the two organs are closely correlated. Forty-one targets were involved in the most highly related pathways associated with CVDs and liver diseases, which may provide a basis for CVDs and liver diseases treatment strategies as well.

### 2.4. Target-Pathway Network

**Figure 5:**
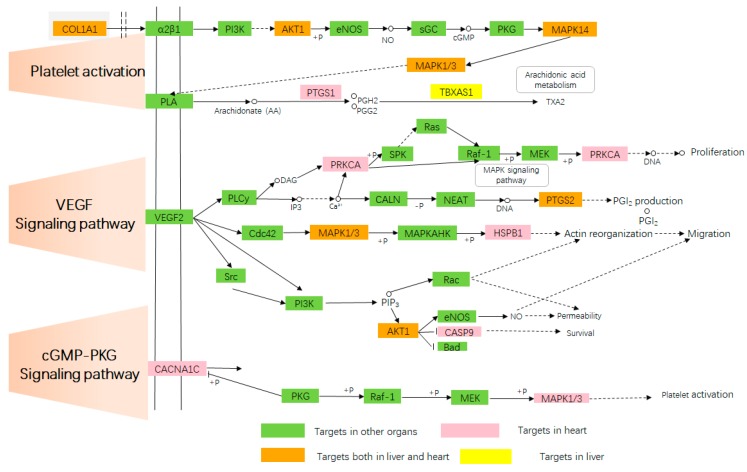
The representative cardiovascular disease pathway. The solid arrow represents molecular interaction or relation (activation), the dashed arrows indirect link or unknown reaction. The circle represents chemical compound, DNA and other moleculee. 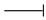: molecular interaction or relation (inhibition). 
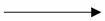
: Phosphorylation, 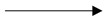: dephosphorylation.

**Figure 6:**
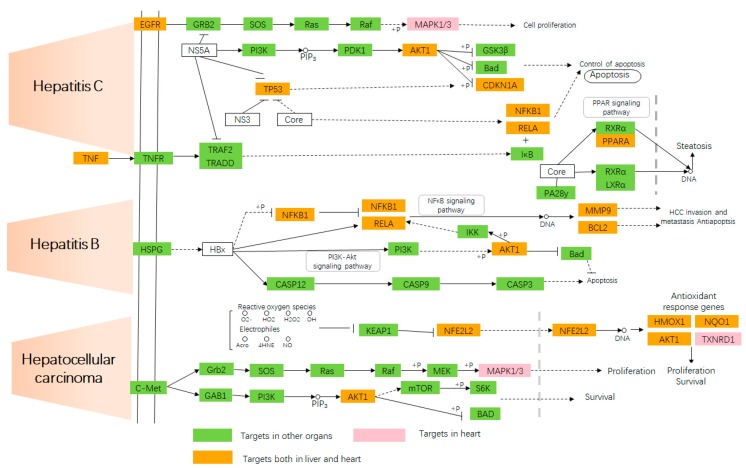
The representative liver disease pathway. The solid arrow represents molecular interaction or relation (activation), the dashed arrows indirect link or unknown reaction. The circle represents chemical compound, DNA and other moleculee. 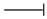: molecular interaction or relation (inhibition). 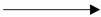: Phosphorylation, 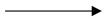: dephosphorylation.

In this section, pathways directly related to CVDs were assembled into a “CVDs pathway” based on the present cognition of CVDs pathology. The mechanisms of pungent flavored compounds treating liver diseases were mapped to the Target-Pathway (T-P) network according to their target’s information. The T-P network could elucidate the function of potential target proteins related to CVDs [135] and liver diseases [136] in the KEGG (Kyoto Encyclopedia of Genes and Genomes) biological pathway. To understand the molecular mechanisms of pungent flavor and liver/heart organ in treating cardiovascular diseases and liver diseases, we mapped the targets onto their related pathways extracted from the KEGG database (www.genome.jp/kegg) [137] and generated a bipartite graph of T-P Network (Figure 5 and Figure 6), in which a compound and a signal pathway were linked if the compound targets on the proteins appeared in the signal pathways.

Similarly, major pathways (111/121) are also modulated with adjusted *p*-values < 0.05, and many of them have been proved as suitable therapeutic pathways for CVDs and liver diseases, such as cGMP-PKG (cyclic guanosine monophosphate-dependent protein kinase) signaling pathway (hsa04022), VEGF (vascular endothelial growth factor) signaling pathway (hsa04370), Platelet activation (hsa04611), NAFLD (hsa04022), Hepatitis B (hsa05161), Hepatitis C (hsa05160), Hepatocellular carcinoma (hsa05225), and drug metabolism–cytochrome P450 (hsa00982). The cGMP-PKG signaling pathway plays a significant role in cardio protection by monitoring cell death and maintaining intracellular acidosis [138]. The cGMP/PKG signaling pathways are involved in platelet inhibition [139]. The cGMP-PKG signaling pathway was first described to unfold vascular effects by mediating smooth muscle relaxation [140] but was then also linked to beneficial myocardial effects by attenuating hypertrophy and pathological remodeling [141]. Platelets play an important role in hemostasis and clot formation [142]. Platelets are involved in the early phases of liver regeneration [143]. Platelet activation is a complex process that involves different cellular signaling pathways [144]. Platelets play a key role in many physiological functions especially in hemostasis and wound healing processes to maintain the integrity of the circulatory system [145,146]. Increased levels of oxidative stress contribute to the development of atherosclerosis that eventually leads to thrombosis; a principal cause of heart attacks and strokes [144]. The vascular endothelial growth factor (VEGF) signaling pathway (VSP) fulfills a cardinal role in endothelial cells, and its inhibition has a profound cardiovascular impact [147]. VSP inhibitors are used as anti-angiogenic therapies. Generalized endothelial dysfunction predisposes to vasoconstriction, atherosclerosis, platelet activation, and thrombosis (arterial more than venous) [147]. It is generally accepted that VEGF is a major driver of the angiogenic process in physiological and pathological processes in both embryo and adult [148,149]. NAFLD (hsa04022), Hepatitis B (hsa05161), Hepatitis C (hsa05160), Hepatocellular carcinoma (hsa05225) and Drug metabolism—cytochrome P450 (hsa00982) been shown to be highly relevant to liver diseases. These results illustrated pungent flavored compounds could treat cardiovascular diseases and liver diseases by acting on the heart/liver organ from the level of the biological pathway.

## 3. Discussion

### 3.1. The Material Base of Pungent Flavor of Pungent TCMs

We first proposed that spice compounds from TCMs were considered as the material base of pungent flavor with pungent taste or smell. Not only do humans use spices, but also animals could use spices by nature. A study reveals that tiny birds on the French island of Corsica may decorate nests with sweet-smelling herbs to create a pest-free environment [48]. The birds incorporate fragments of 10 or more fragrant plants including yarrow, lavender, mint, and lemon balm into their nests. Maybe they can naturally distinguish the spices with smell or taste.

Spices are always pleasing with their color, flavor or pungency. Spices have been used for thousands of years to enhance the flavor, color, and aroma of food [49]. In addition, they are known for their preservative and medicinal value [49,50,150]. In the past three decades, it has been experimentally documented that several common spices can also exert beneficial health physiological effects [51]. The antioxidant properties of herbs and spices are of particular interest given the impact of oxidative modification of low-density lipoprotein cholesterol in the development of atherosclerosis [52]. Consumption of garlic or garlic oil has been associated with a reduction in total cholesterol, low-density lipoprotein (LDL) cholesterol, and triglyceride levels58. Studies suggest that an intake of between half and one garlic clove per day can reduce cholesterol by 9% [53,54].

### 3.2. Pungent Compounds Provide Support for the Treatment of Cardiovascular and Liver Diseases

Pungent traditional Chinese medicine provides abundant resources of pungent compounds with a fragrant smell and pungent taste. The authors believe that the pungent compounds with pungent taste or aromatic odor from pungent TCMs are the material base of pungent flavor in TCM terms, and they have the potential action of blood-activating and stasis-resolving. These compounds are from natural products and have good bioavailability and non-toxic effects. Moreover, the pungent compounds are associated with undesirable effects on the cardiovascular system and temperature. These compounds could become a potential drug resource in treating cardiovascular and liver diseases.

### 3.3. Expression of Meridian Tropism at Level of Tissue and Organ

These traditional Chinese medicines often exhibit high similarity in their TCM herbal properties (TCM-HPs). The classic concept of TCM-HPs defines four fundamental characters (cold, cool, warm, and hot), five fundamental flavors (salty, sour, bitter, sweet, and pungent), four toxic states (toxic, nontoxic, very toxic, and slightly toxic), 12 meridians (bladder, spleen, large intestine, stomach, small intestine, liver, lung, heart, kidney, gallbladder, xin bao or pericardium and san jiao) [11,151,152]. TCM-HP is the core of basic theory of TCM and is the high recapitulation of clinical practice from ancient physicians [11]. They described TCMs from multiple perspectives (character, flavor, toxic, meridian, etc.), but due to the limitation of the medical level at that time, the scientific connotation of TCMs could not be explained at the molecular level. TCM-HP also provides strong evidence to guide the clinical application of TCM. From the view of modern science, four fundamental characters reflect the characters of TCMs, five fundamental flavors reflect the properties of TCMs, and meridians reflect human body parts which the TCMs act on. The TCM entering one meridian or several meridians means the corresponding efficacy of such herb has obvious action towards this or these organs/meridians with no obvious or even no effect on other parts [11].

Traditional Chinese medicine believed that the cardiovascule was deficiency in the “root” and excessed in the “branch”. Its “root” was on liver and its “branch” was on heart. In the long-term clinical practice, the theory of treating cardiovascular diseases from the liver was gradually developed. The theory stems from Huangdi Neijing (The Yellow Emperor’s Inner Canon) [153]. In traditional Chinese Medicine, the human body is considered as a holistic being in which each organ or each specific physiological part is interrelated. Based on Huangdi Neijing (The Yellow Emperor’s Inner Canon) [153], traditional Chinese medical practitioners diagnose and treat patients beneath the guidance of meridian theory, declaring that the liver and heart linked through some specific Jingluo (meridians) [154], and they have an interaction effect in terms of both physiological function and pathological basis. As for physiological function, the heart is able to control the blood and vessels and govern the mind [135], while the liver stores blood, controls conveyance and dispersion, dominates the tendons, has its outward manifestation in the nails, and opens into the eyes.

However, the relationships between five flavors (the true taste or smell) and meridian tropism are not explicit. Additionally, the underlying molecular mechanisms of pungent flavor and liver meridian tropism in treating CVDs and liver diseases are yet unclear.

These five TCMs are with common traditional Chinese medicine herbal properties (TCM-HPs), namely warm, pungent, and liver meridian tropism. They all have the single pungent flavor and belong to the same liver meridian tropism. We will focus on the relationship between pungent flavor and the liver meridian tropism at the molecular level in treating cardiovascular diseases and liver diseases.

## 4. Materials and Methods

### 4.1. Date Collection and Visualization

In this work, the available information of traditional Chinese medicine herbal property (TCM-HP) was extracted from pharmacopoeia of the People’s Republic of China (2015) [155], which consists of 5608 different species of drugs in the texts, such as herbs, plant oils, and extracts.

In this study, the blood-activating and stasis-resolving (BASR) TCMs of *Chuanxiong Rhizome* (Chinese name Chuanxiong), *Carthami Flos* (Chinese name HongHua), *Dalbergiae Odoriferae Lignum* (Chinese name JiangXiang), *Foeniculi Fructus* (Chinese name XiaoHuiXiang), and *Angelicae Sinensis Radix* (Chinese name DangGui) were selected as the research object for pungent flavor. These five BASR TCMs have the single pungent flavor and belong to the same liver meridian tropism [11,54,151]. 

**Figure 7:**
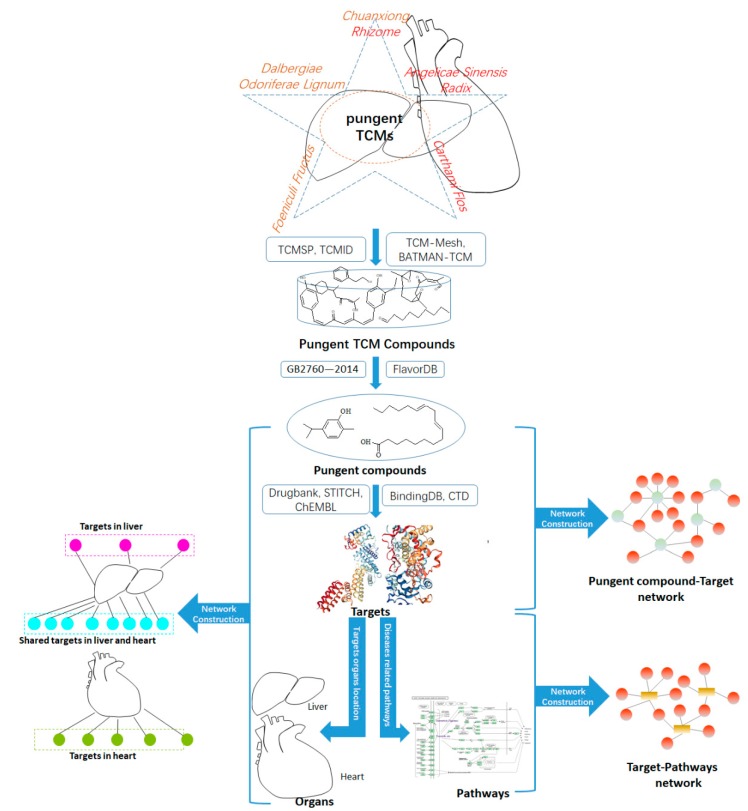
The workflow to study the relationship between pungent flavored compounds and their special targets organ location in the cardiovascular system. The cyan circle represents the shared target in liver and heart. The magenta circle represent the targets in liver. The yellowish green circle represents the target in heart.

A systems pharmacology approach was used to depict pungent compounds in treating CVDs and liver diseases from the molecular to holistic level. Briefly, as shown in Figure 7, we collected the compounds of pungent TCMs from TCMSP [156], TCMID [45,46], BATMAN-TCM [157], TCM-Mesh [158], TCM Database@Taiwan [159]. All of these compounds collected were normalized to the canonical SMILES format [160]. Duplicates from different sources and compounds without structures were excluded. The PaDEL-Descriptor [161] was used to generate a set of MDL (Molecular Design Limited) 166-key fingerprints for each TCM compounds. The MDL 166-key fingerprint is also known as the “Molecular Access System (MACCS)” key in the literature and is a no hashed fingerprint consisting of 166 bits. The MDL 166-key fingerprint is one of the very few availabilities that offers a 1-1 mapping. These fingerprints were assembled into the fingerprint array. 

The potential related targets of pungent flavor compounds were from drugbank [162,163], STITCH [164], ChEMBL [165], BindingDB [166,167]. The obtained targets were mapping into relevant databases to find out their corresponding pathways of CVDs and liver diseases. Furthermore, network construction, pathway enrichment analysis, tissue location, and organ location analysis were performed to illustrate the molecular mechanisms of pungent compounds treating CVDs and liver diseases holistically.

**Figure 8:**
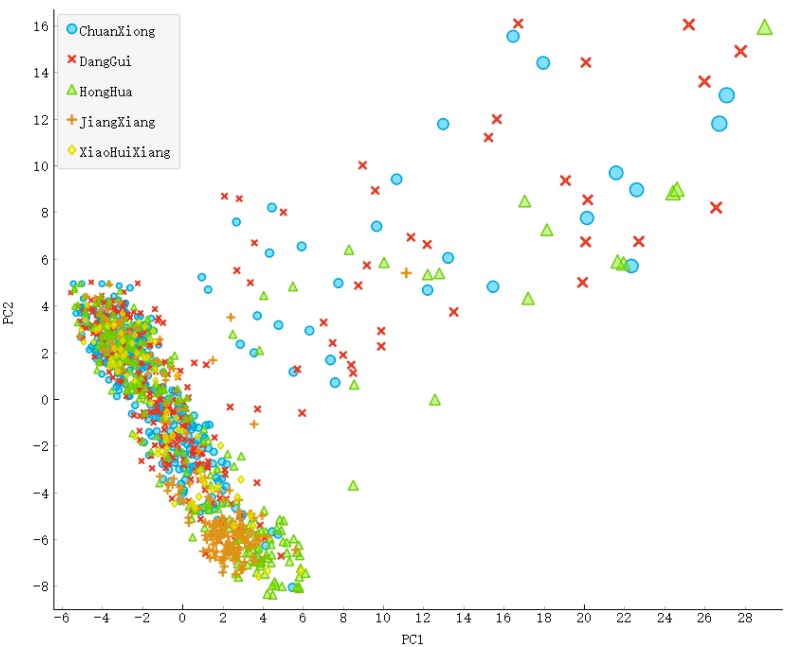
Five pungent TCMs’ compounds PCA with Fingerprint of 166 bits.

We collected 1159 compounds of 5 pungent TCMs (Table 5) and then generate a set of MDL 166-key fingerprints for each compounds’ with PaDEL-Descriptor [161]. The 1159 compounds from 5 pungent TCMs could be mapped into two-dimensional space with the PCA (Principal Component Analysis) method while maintaining a lot of geometric structure (Figure 8). 

It can be seen from Figure 8 that the structural formulae of these five pungent TCMs were similar, indicating that the five pungent TCMs have the same or similar efficacy. Nevertheless, there are many compounds with similar structure in these TCMs.

The pungent flavor compounds of pungent TCMs were identified from spice [168] with FEMA code [169] and flavorDB [170], which consisted of 1511 different spices and pungent flavor compounds in total.

The diversity of the pungent TCMs’ compounds molecules within each TCM was computed as follows [171]. Let $d_{\mu \nu }$ be the Soergel-based [172] interdata set distance between the two TCMs’ compounds sets $D_{\mu }$ and $D_{\nu }$.

(1) $$d_{\mu \nu }=\frac{1}{N_{\mu }N_{\nu }}\sum_{i=1}^{N_{\mu }}\sum_{j=1}^{N_{\nu }}d_{sg}\left(x_{i}^{\mu },x_{j}^{\nu }\right)$$

where $N_{\mu }$ and $N_{\nu }$ are the number of molecules in TCM compound data set $D_{\mu }$ and $D_{\nu }$, and $x_{\gamma }^{s}$ is the fingerprint vector from rowγof the fingerprint array for data set $D_{s}$. Let $d_{\mu }$ be the diversity of the molecules within a single data set $D_{\mu }$ as Equation (1) [172].

(2) $$d_{sg}=\frac{\sum_{k=1}^{d}\left|P_{k}-Q_{k}\right|}{\sum_{k=1}^{d}\max\left(P_{k}-Q_{k}\right)}$$

where $P_{k}$ and $Q_{k}$ are the fingerprint vector of two compounds in each pungent TCM compounds set. 

The compounds data set of *Angelicae Sinensis Radix* (DangGui) has the greatest diversity (Table 2), so this data set is expected to exhibit the weakest clustering, and this pungent TCM has more extensive potential pharmacological effects.

### 4.2. The Gene Analysis of Liver and Heart Organ in Cardiovascular System 

The targets of pungent flavor compounds associated with CVDs and liver diseases were obtained from literature mining and several disease-gene databases: DrugBank (http://www.drugbank.ca/) [162,163], STITCH (http://stitch.embl.de/) [164], ChEMBL (https://www.ebi.ac.uk/chembl/) [165], BindingDB (http://www.bindingdb.org/bind/index.jsp) [166,167]. Therapeutic Target Database (http://bidd.nus.edu.sg/group/ttd/) [173,174], and Comparative Toxicogenomics Database (CTD, http://ctdbase.org/) [175]. 

#### 4.2.1. Identify the Pungent Flavored Compounds

The process of discovering the material basis of the pungent flavored TCMs can be clearly seen from Figure 1. The pungency is attributable to chemicals that activate a specific transient receptor potential (TRP) cation channel family. Moreover, the ligands of TRPs are most spices and flavor molecules. So, the spices flavor molecules were considered as the materials base of pungent flavor. The pungent flavored compounds of pungent TCMs are from Food Safety National Standard for use of food additives (GB2760-2014) [117] and flavorDB. The GB2760-2014 is the National Standard of Spice. FlavorDB [119] (http://cosylab.iiitd.edu.in/flavordb) comprises of 25,595 flavor molecules representing an array of tastes and odors. Among these, 2254 molecules are associated with 936 natural ingredients belonging to 34 categories. Molecules from the chemical basis of flavor expressed primarily via gustatory and olfactory mechanisms [119]. The simple perception of pungent TCMs arises from the interaction of flavor molecules with the biological machinery by ancient medical practitioners’ human sensory system. Flavors derived from TCMs have shaped the pungent flavor throughout ancient long-term clinical practice in China.

#### 4.2.2. Identify Organ Target Location with Gene Organizer

The herb entering one meridian, or several meridians means the corresponding efficacy of such herb has obvious action towards this or these organs or meridian but no obvious effect to other parts. Due to the limitation of repertoire of expression datasets, there is a strong bias towards certain organs and tissues (e.g., brain, blood, and skin), and many other body parts are rare or completely absent. TCM theory has always focused on the interaction of TCMs with human body on a holistic view. Gene ORGANizer [134] was a comprehensive and fully curated Database, consisting of >150,000 gene-body part associations, and covering over 7000 human genes. So, Gene ORGANizer provides the strong evidence for elucidating the connotation of meridian tropism at the target level. The 5 pungent TCMs were all attributable to liver tropism. Moreover, among these pungent TCMs, *Carthami Flus* (Chinese name HongHua) and *Angelicae Sinensis Radix* (Chinese name DangGui), *Chuanxiong Rhizome* (Chinese name ChuanXiong) also belong to the heart or pericardium meridian tropism. The targets of pungent TCMs’ pungent flavored compounds were mapped into the organ location map at the cardiovascular system level by Gene ORGANizer. We analyzed the correlation of genes associated with liver organ and heart organ based on phenotype. 

### 4.3. Network Construction

To further elucidate multi-scale action mechanisms of pungent TCMs in the prevention and treatment of CVDs and liver diseases, we constructed two networks: Pungent Compound-Target network (C-T network) and Target-Pathway network (T-P network). In the network, the nodes represent compounds/targets/pathways, and edges represent they are linked with each other. The canonical pathways were extracted from the KEGG database (http://www.genome.jp/kegg/) [137,176]. The enriched KEGG pathways of targets with a false discovery rate of less than 0.005 by Fisher’s Exact test in the DAVID database (https://david.ncifcrf.gov/) [177] were analyzed. In these networks, degree (DD) is used to characterize the connectedness of a node. The degree of a node is the number of edges associated with it. The networks were generated and analyzed by using Cytoscape 3.2.1 [178]. The topological properties of these networks were analyzed using the Network Analysis plugin CentiScaPe 1.2 of Cytoscape [179]. The degree of a node was defined as the number of edges connected to it, implying the importance of the node in a network.

## 5. Conclusions

In this study, we first proposed a novel strategy integrating target organ location, five flavor theory, and systems pharmacology approach to explore the molecular mechanism of pungent flavored compounds from pungent BASR TCMs in the synchronic treatment for cardiovascular diseases and liver diseases by acting on the heart and liver organ. Our main findings are as follows:

(1) The spice compounds were considered as the material basis of pungent flavor and their holistic efficacy at the special organ level. 

(2) We systematically analyzed the relationship between pungent flavored compounds in pungent TCMs and their targets’ organ location map.

(3) A novel system is constructed to investigate the closeness between targets of liver and heart in the cardiovascular system. The shared targets of liver and heart were identified, and the association was evaluated.

In summary, this study provided a systematic analysis of relationship between pungent compounds from pungent TCMs and the holistic efficacy of their targets in treating CVDs and liver diseases. We found potential active compounds to treat CVDs and liver diseases by the system pharmacology approach and understand the molecular mechanisms of how these 5 pungent TCMs treat CVDs and liver diseases by acting on heart organ and liver organ. Despite these potentially interesting findings above, further interpretation, such as the functional pungent flavor in TCMs and the herb dose-effect relationship, is necessary to be considered based on experimental data analysis. Moreover, further experimental testing of these pungent flavored compound-target binding actions and molecular mechanism of pungent flavored compounds from pungent TCMs in vivo will be required to support further assessments of potential clinical application.

## Figures and Tables

**Table 1:** The flavor of some pungent traditional Chinese medicines (TCMs).

Latin Name	English Name	Chinese Name	Flavor
*Piper Nigrum*	Black pepper	Heihujiao	Fragrant smelling, taste pungent
*Syzygium aromaticum*	Cloves	Dingxiang	Strong fragrant smelling, taste pungent and feeling on tongue
*Trigonella foenum-graecum L.*	Fenugreek	Huluba	Sweet smelling aroma; fragrant
*Menthae Haplocalycis Herba*	Peppermint	Bohe	Cool and fragrant smelling, taste pungent
*Zanthoxyli Pericarpium*	Pepper	Huajiao	Fragrant smelling; taste sweet and pungent
*Myristicae Semen*	Nutmeg	Roudoukou	Strong fragrant smelling, taste pungent
*Aucklandiae Radix*	Common vladimiria root	Muxiang	Specific fragrant smelling, taste slightly bitter
*Origanum vulgare L.*	Oregano	Niuzhi	Taste pungent and slightly bitter
*Thymus mongolicus* *Ronn*	Thyme	Bailixiang	Taste pungent
*Cinnamomt Cortex*	Cinnamon	Rougui	Strong fragrant smelling, taste sweet and pungent
*Rosmarinus officinalis*	Rosemary	Midiexiang	Pungent
*Allii Sativi Bulbus*	Garlic	Dasuan	Specific smelling, taste pungent
*Zingiberis Rhizoma Recens*	Ginger	Shengjiang	Specific fragrant smelling, taste pungent
*Alpiniae Officinarum Rhizoma*	Galangal	Gaoliangjiang	Fragrant smelling, taste pungent

**Table 2:** Data sets on pungent compounds to evaluate structure clustering.

*Latin Name*	Chinese Herb	Diversity	No. Entries	Descriptor
*Chuanxiong Rhizome*	ChuanXiong	0.6678	352	TCMID, TCMSP, BATMAN-TCM, TCM Database@Taiwan, TCM-Mesh
*Carthami Flos*	HongHua	0.7011	272	
*Dalbergiae Odoriferae Lignum*	JiangXiang	0.5255	169	
*Foeniculi Fructus*	XiaoHuiXiang	0.6495	74	
*Angelicae Sinensis Radix*	DangGui	0.7116	292	

**Table 3:** Pungent flavored compounds from pungent TCMs.

Latin Name	Herbs	Molecule Name	FEMA or PubChem ID
*Chuanxiong Rhizome*	ChuanXiong	Ethylpalmitate	2451
		Trimethylamine	3241
		Vanillin	3107
		Nonanal	2782
		1,8-cineole	2465
		Myrcene	2762
		Methyleugenol	2475
		hexanal	2557
		thymol	3066
		*trans*-2-Nonen-1-ol	3379
		P-Cymene	2356
		Borneol	2157
		Naphthalene	pubchem_931
		Phenylacetaldehyde	2874
		O-Cresol	3480
		Linalool	2635
		Camphene	2229
		Linoleic Acid	3380
		3-Carene	3821
		2-pentylfuran	3317
*Angelicae Sinensis Radix*	DangGui	Camphene	2229
		Carvacrol	2245
		o-Cresol	3480
		p-Cresol	2337
		p-Ethylphenol	3156
		Guaiacol	2532
		Isoeugenol	2468
		Phenol	3223
		Vanillin	3107
		Nonanal	2782
		Myrcene	2762
		naphthalene	pubchem_931
		1-hexadecanol	2554
		m-cresol	3530
		3-carene	3821
*Carthami Flos*	HongHua	Benzothiazole	3256
		3-Hexanol	3351
		Phenylacetaldehyde	2874
		Nonanal	2782
		Methylcinnamate	2698
		carvacrol	2245
		Isovalericacid	3102
		stearicacid	3035
*Dalbergiae Odoriferae Lignum*	JiangXiang	1,8-cineole	2465
		indole	2593
		Ethylpalmitate	2451
*Foeniculi Fructus*	XiaoHuiXiang	D-Limonene	2633
		α-Terpinene	3558
		Terpinolene	3046
		1,8-cineole	2465
		Myrcene	2762
		acetaldehyde	2003
		d-fenchone	2479

**Table 4:** TOP 20 Targets information of pungent TCMs in the cardiovascular system.

Target	UniProt ID	Gene Name	Related Diseases	Degree
*NFE2L2*	Q16236	Nuclear factor erythroid 2-related factor 2	Atherosclerosis, hypertension, Heart failure	16
*AR*	P10275	Androgen Receptor	Atherosclerosis, Coronary artery disease, Hypertension, Myocardial infarction	15
*PTGS2*	P35354	Prostaglandin G/H synthase 2	Pain, Coronary heart disease, Myocardial infarction, Vascular lesion regression	13
*TNF*	P01375	Tumor necrosis factor	Heart failure, Hepatitis C infection, Hypertension, Coronary atherosclerosis, Cirrhosis	13
*NFKB1*	P19838	Nuclear factor NF-kappa-B p105 subunit	Atherosclerosis, Cancer, Type 2 diabetes, Hepatitis C, Chronic Liver cirrhosis	12
*PPARD*	Q03181	Peroxisome proliferator-activated receptor delta	Central nervous system disease, Metabolic disorders, Atherosclerosis, Coronary; Coronary heart disease	12
*CYP1A1*	P04798	Cytochrome P450 1A1	Liver cancer; Liver disease, Coronary artery disease, Hypertension	12
*JUN*	P05412	Proto-oncogene c-JUN	Heart failure, Breast cancer	11
*CYP2E1*	P05181	Cytochrome P450 2E1	Liver cancer; Liver disease	10
*AHR*	P35869	Aryl hydrocarbon receptor	Multiple myeloma, HIV infections, Myocardial infarction	10
*CAT*	P04040	Catalase	Skin burns, Heart failure	10
*ALB*	P02768	Serum albumin	Hemophilia’ Visualizing lesions with abnormal blood brain barrier	10
*PTGS1*	P23219	Cyclooxygenase-1	Platelet function, Myocardial Infarction, Acute coronary syndrome	9
*CXCL8*	P10145	Interleukin-8	Melanoma. Autoimmune diabetes	9
*MMP9*	P14780	Matrix metalloproteinase 9	Atherosclerosis, Carotid, Colorectal cancer	9
*HMOX1*	P09601	Heme oxygenase 1	Myocardial infarction, Atherosclerosis, Coronary, Cardiovascular disease, Ischemic injury of the liver, Inflammation, Cerebral vasospasm	9
*IL1B*	P01584	Interleukin-1 β	Coronary disease, Hypertension, Chronic hepatitis C	8
*IL6*	P05231	Interleukin-6	Arteriosclerosis, Heart valve diseases, Hepatitis B	8
*ACE*	P22303	Acetylcholinesterase	Chronic heart failure, Hypertension	8
*HSPB1*	P04792	Heat shock protein β-1	Breast cancer. Ovarian cancer, Bladder cancer, Prostate cancer, Lung cancer, Arthritis	8

**Table 5:** The details of 5 pungent TCMs in this research.

*Latin Name*	Chinese Herb	TCM-HPs
*Chuanxiong Rhizome*	ChuanXiong	Warm, pungent, liver, gallbladder, pericardium meridian tropism
*Carthami Flus*	HongHua	Warm, pungent, liver, heart meridian tropism
*Dalbergiae Odoriferae Lignum*	JiangXiang	Warm, pungent, liver, spleen meridian tropism
*Foeniculi Fructus*	XiaoHuiXiang	Warm, pungent, liver, kidney, spleen, stomach meridian tropism
*Angelicae Sinensis Radix*	DangGui	Warm, pungent, liver, heart, spleen meridian tropism

## Abbreviations

BASR	blood-activating and stasis-resolving
cGMP-PKG	cyclic guanosine monophosphate-dependent protein kinase
CNS	central nervous system
CVDs	cardiovascular diseases
CYP1A1	cytochrome P450 1A1
JUN	proto-oncogene c-JUN
KEGG	Kyoto Encyclopedia of Genes and Genomes
LDL	low-density lipoprotein
MACCS	molecular Access System
MDL	Molecular Design Limited
NAFLD	non-alcoholic fatty liver disease
NFE2L2	nuclear factor erythroid 2-related factor 2
NFKB1	nuclear factor NF-kappa-B p105 subunit
PCA	Principal Component Analysis
PPARD	peroxisome proliferator-activated receptor delta,
PTGS2	prostaglandin G/H synthase 2
TCM	traditional Chinese medicine
TCM-HPs	traditional Chinese medicine herbal properties
TNF	tumor necrosis factor
TPAs	target-pathway association interactions
TRP	transient receptor potential
TRPA1	transient receptor potential ankyrin 1
TRPM7	potential cation channel, subfamily M, member 7
TRPM8	potential cation channel, subfamily M, member 8
TRPV1	transient receptor potential cation channel subfamily V member 1
TRPV3	transient receptor potential cation channel subfamily V member 3
VEGF	vascular endothelial growth factor
